# Maraviroc, a CCR5 Antagonist, Prevents Development of Hepatocellular Carcinoma in a Mouse Model

**DOI:** 10.1371/journal.pone.0053992

**Published:** 2013-01-09

**Authors:** Laura Ochoa-Callejero, Laura Pérez-Martínez, Susana Rubio-Mediavilla, José A. Oteo, Alfredo Martínez, José R. Blanco

**Affiliations:** 1 Oncology Area, Center for Biomedical Research of La Rioja (CIBIR), Logroño, Spain; 2 Infectious Diseases Area, Center for Biomedical Research of La Rioja (CIBIR), Logroño, Spain; 3 Pathology Service, Hospital San Pedro, Logroño, Spain; Mount Sinai School of Medicine, United States of America

## Abstract

Chronic liver disease may result in a sequential progression through fibrosis, cirrhosis and lead, eventually, to hepatocellular carcinoma (HCC). Hepatic stellate cells (HSC) seem to be responsible for the fibrogenic response through the activation of an autocrine loop involving the chemokine receptor, CCR5. However, the role of CCR5 in HCC remains poorly understood. Since this receptor is also one of the main ports of entry for the human immunodeficiency virus (HIV), several CCR5 inhibitors are being used in the clinic to reduce viral load. We used one of these inhibitors, maraviroc (MVC), in a mouse model of diet-induced HCC to investigate whether this intervention would reduce disease progression. Animals treated with MVC on top of a normal control diet did not present any evidence of toxicity or any morphological change when compared with non-treated mice. Animals treated with MVC presented higher survival, less liver fibrosis, lower levels of liver injury markers and chemokines, less apoptosis, lower proliferation index, and lower tumor burden than their counterparts receiving only the hepatotoxic diet. In addition, MVC inhibits HSC activation markers such as phosphorylation of p38 and ERK, and increases hepatocyte survival. This study suggests that MVC, a well tolerated and clinically characterized drug, may be used as a preventative treatment for HCC. Clinical studies are needed to demonstrate the efficacy of this drug, or other CCR5 inhibitors, in patients with high risk of developing HCC.

## Introduction

Liver disease is an important cause of mortality in the world and its incidence is increasing, unlike other major causes of mortality [1]. Hepatocellular carcinoma (HCC) accounts for approximately 6% of all new cancer cases diagnosed worldwide. Liver cancer is the fifth most common cancer among men worldwide, and the eight in women. Geographically, 83% of all cases appear in developing countries [2]. Globally, the etiology of HCC is dominated by the interaction of viral and environmental risk factors. Epidemiological and experimental evidence demonstrate the carcinogenic effect of chronic infection with hepatitis viruses B (HBV) and C (HCV). Worldwide, the proportion of HCC attributable to chronic hepatitis is about 54% for HBV and 31% for HCV. Dietary exposure to aflatoxins in low-resource tropical countries is a significant risk factor that operates synergistically with hepatic infections [3]. In developed countries, the main concomitant risk factors are obesity and metabolic syndrome, smoking, and chronic alcohol abuse [4].

Currently, treatment of HCC is restricted to surgical resection or liver transplant, but only 20% of patients can be subjected to these procedures [5]. Prevention is always the best strategy to reduce liver cancer, especially through hepatitis vaccination and aflatoxin removal campaigns [6] but little can be done once chronic disease is rampant. Few specific chemotherapeutic options are available for this cancer; one of these being sorafenib [7]. Therefore, new therapeutic approaches are urgently needed.

Regardless of etiology, chronic liver disease generally involves a process of progressive destruction and regeneration of the liver parenchyma, leading to fibrosis and cirrhosis. At early stages most patients are asymptomatic and can easily go undiagnosed and untreated for decades [8]. This chronic liver injury is characterized, at the molecular level, for the rapid turnover and excessive accumulation of extracellular matrix proteins which replace the functional parenchyma by fibrotic tissue [9]. Hepatic stellate cells (HSC) are the main source of the fibrotic tissue and, upon chronic damage, they secrete numerous inflammatory mediators including chemokines CCL3, CCL4, and CCL5, among others [10], [11]. Simultaneously, HSC express several chemokine receptors such as CXCR3, CCR1, CCR3, CCR5, and CCR7 [12], [13]. Moreover, HSC express the other HIV co-receptor, CXCR4. Binding of this receptor by its endogenous ligand, CXCL12, also has pro-fibrogenic effects on HSC [14]. It seems that the paracrine and autocrine activation of these receptors promotes the fibrogenic response [15], which is characterized by increased collagen synthesis, impaired collagen degradation, and secretion of further inflammatory mediators [16]. The progressive fibrosis and persistent liver inflammation would eventually lead to HCC [17].

CCR5 plays a central role in all the events related to liver matrix remodelling and it has been observed that patients with chronic liver disease present high levels of CCR5 and CCL5 [18]. In addition, gene targeting or the use of a potent antagonist for the murine CCR5 receptor results in a significant reduction of liver fibrosis [16], [18]. Interestingly, CCR5 is also the coreceptor for the most commonly transmitted HIV-1 strains [19]; so several pharmaceutical companies have developed specific small molecule antagonists that are being used as antiviral therapies, but are also effective in blocking CCR5 signal transduction. These include maraviroc (MVC) [20], [21], vicribiroc [22], TBR-652 [23], and INCB9471 [24]. Another inhibitor, aplaviroc, was discontinued due to excessive hepatotoxicity during clinical trials [25]. A natural product antagonist, anibamine, is currently undergoing preclinical characterization [26].

If these antagonists block CCR5 signalling, we hypothesized they should prevent the consequences of activating the receptor, such as liver fibrosis and all the downstream manifestations including HCC. In fact, there is some preliminary evidence that HIV patients coinfected with HCV that received MVC to reduce their HIV load, benefited from a reduction in liver stiffness [27].

To demonstrate whether CCR5 inhibitors prevent HCC, we used a mouse model where the animals are exposed to a choline-deficient diet supplemented with ethionine in the drinking water (CDE) [28], [29]. This model has the advantage of recapitulating most of the stages of the human disease, progressing from liver damage to fibrosis, and finally HCC [30]. We found that treatment of these animals with MVC greatly reduced mortality, markers of liver damage, apoptosis, proliferation, expression levels of chemokines, fibrosis, and hepatic tumor load.

## Results

### MVC Improved Survival of CDE-treated Animals

**Figure 1:**
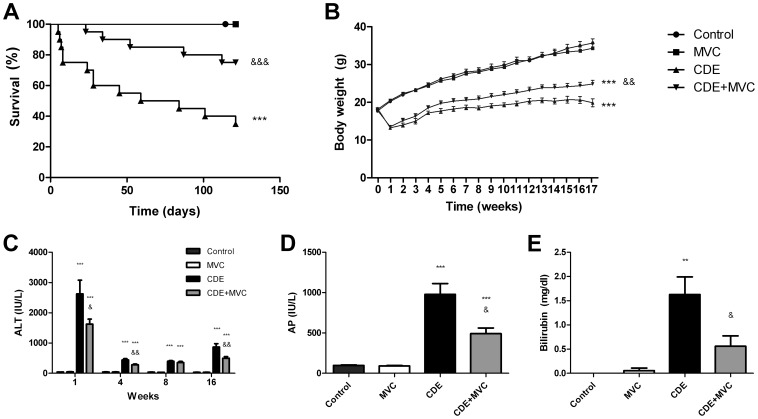
Survival, body weight, and liver damage markers. A Kaplan-Meier survival plot shows that no deaths occurred in Groups Control or MVC. In Groups CDE and CDE+MVC deaths were registered along the time of the experiment (**A**). Mean survival was 92 days for Group CDE whereas all other Groups did not reach that parameter. There were no statistically significant differences between Group CDE+MVC and those not receiving the CDE diet (Control and MVC). There was a very significant difference in survival between Group CDE and any of the other Groups. When body weight was measured (**B**), Groups which received the CDE diet (CDE and CDE+MVC) displayed a serious weight loss in the first week. This parameter recovered slowly in the following weeks. Nevertheless, the animals that were treated with the CCR5 inhibitor (CDE+MVC) recovered weight at a significantly higher rate than non treated animals (CDE). The markers of liver damage studied included transaminases (**C**), alkaline phosphatase (**D**), and bilirubin (**E**). Transaminase blood levels were measured at 4 time points during the experiment, whereas levels of AP and bilirubin were only measured at the end of the procedure (week 16). There was an abrupt increase of transaminases in animals that received the CDE diet during the first week, which diminished later but never reached the basal levels observed in the control diet Groups. Mice that received the CCR5 inhibitor (CDE+MVC) had significantly lower levels of transaminases than those who did not (CDE). The same pattern was observed for AP and bilirubin. Each bar represents the mean ± SEM of at least 8 animals. **p<0.01; ***p<0.001 with respect to control; &p<0.05; &&p<0.01; &&&p<0.001 with respect to CDE.

Previous studies have shown that CDE treatment causes acute inflammation of the liver and some animals die shortly after beginning the diet [30], [31]. In our experiment, all animals assigned to Groups Control and MVC remained healthy throughout the duration. In clear contrast, numerous deaths were recorded in Group CDE, especially during the first week, although more deaths occurred later at lower frequency. Interestingly, the number of deaths in Group CDE+MVC was much smaller than in Group CDE (Fig. 1A). Statistical analysis of these survival data showed very significant differences between Group CDE and any of the other treatments (p<0.001) whereas no significant differences were found between Group CDE+MVC and the control Groups Control and MVC (p>0.05). Cox’s regression analysis indicated that mice in Group CDE had a 3.7-fold higher chance of dying than those in Group CDE+MVC (95% CI: 1.3–10.6).

### Mice Treated with MVC Recover Better from CDE Diet-induced Weight Loss

Body weight was measured weekly (Fig. 1B). Animals in Groups Control and MVC followed a normal growth pattern and no differences between them were observed. On the other hand, animals treated with the CDE diet (Groups CDE and CDE+MVC) suffered a steep weight loss on the first week, followed by a slow recovery during the next weeks. The growth rate of these animals was always slower than that of the control Groups (Groups Control and MVC). Nevertheless, mice in Group CDE+MVC (treated with MVC) had a recovery rate significantly higher than that of Group CDE (p<0.01).

### MVC Reduced Liver Damage

High levels of circulating transaminases, alkaline phosphatase (AP), or bilirubin indicate the existence of liver damage [32]. To study liver function modifications produced by the diets, alanine aminotransferase (ALT) levels were measured in the 4 experimental Groups at 1, 4, 8, and 16 weeks after diet initiation, and AP and bilirubin were measured at the time of sacrifice (16 weeks). As expected, Groups Control and MVC had no detectable levels of these injury markers, whereas Groups CDE and CDE+MVC presented high levels of ALT at 1 week that recovered slowly after. Remarkably, statistically significant differences were found between the ALT levels measured in Group CDE and CDE+MVC, at 1 week (p<0.05), 4 weeks (p<0.01), and 16 weeks (p<0.01), with Group CDE+MVC having lower ALT levels than Group CDE (Fig. 1C). Moreover, the levels of AP (Fig. 1D) and bilirubin (Fig. 1E) were significantly higher in Group CDE than in Group CDE+MVC, indicating that MVC reduces jaundice and bile duct obstruction.

### MVC Prevented CDE Diet-induced Hepatomegaly and Splenomegaly

**Figure 2:**
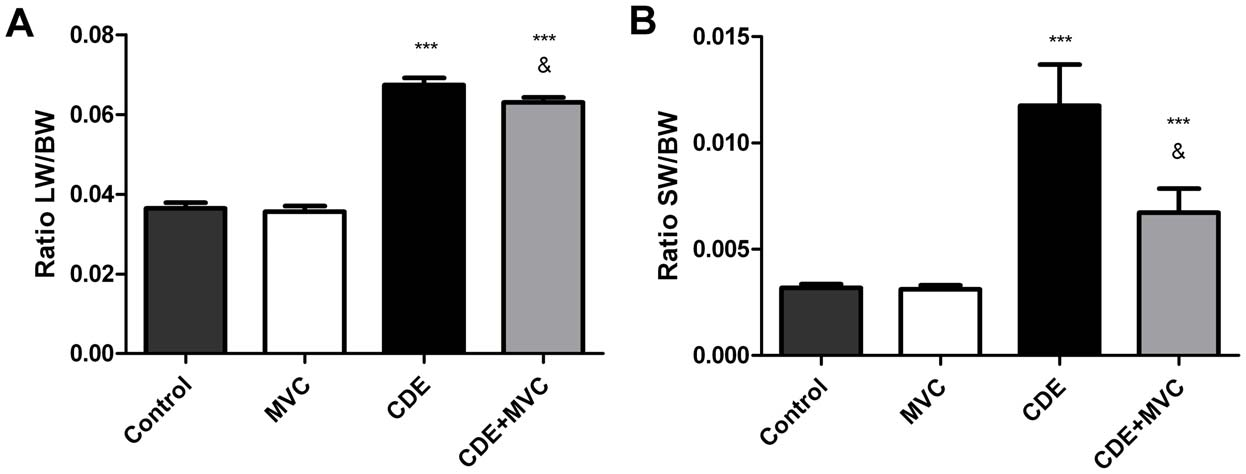
Relative weight of the liver and the spleen. A significant increase in the relative weight (weight of the organ divided by the body weight) of both organs was recorded in the animals receiving the CDE diet, compared with the control diet. Among the mice that received the CDE diet, those treated with MVC had a significantly smaller liver (**A**) and spleen (**B**) than those who were not treated. ***p<0.001 with respect to control; &p<0.05 with respect to CDE.

At the time of sacrifice, the liver and spleen of experimental animals were weighted. There was a significant increase in liver weight in the animals receiving the CDE diet (Groups CDE and CDE+MVC) when compared with those receiving a control chow (Group Control and MVC). Liver weight was significantly lower (p<0.05) in Group CDE+MVC than in Group CDE (Fig. 2A). With the spleen, the results were similar, finding splenomegaly in animals belonging to Groups CDE and CDE+MVC. In this case, as well, the spleen of mice in Group CDE+MVC was significantly smaller (p<0.05) than those in Group CDE (Fig. 2B).

### MVC Prevented CDE Diet-induced Carcinogenesis

**Figure 3:**
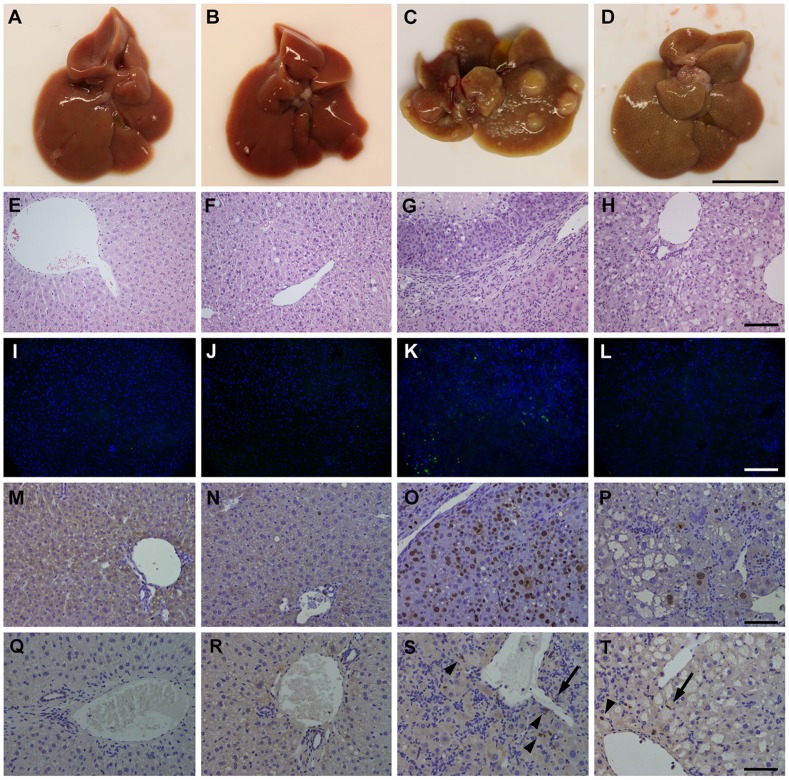
Representative photographs and microphotographs of the liver. A clear change in color and general texture was easily appreciate when comparing the liver of animals treated with control diet (**A,B**) or with the CDE one (**C,D**). The liver of animals in the CDE Group presents a large number of tumors (**C**). Tumors in Group CDE+MVC were less numerous and much smaller than those in the previous Group (**D**). Scale bar for A–D = 1 cm. Histological images were stained with hematoxylin-eosin (**E–H**), with the fluorescent TUNEL technique (**I–L**), anti Ki67 (**M–P**), or with anti-CCR5 antibody (Q–T). The first 2 Groups; Control and MVC (**E,F**) displayed a normal liver morphology. The liver of the CDE Group had numerous atypic cells and frank tumors (**G**). Animals treated with MVC had intermediate characteristics (**H**). Scale bar for E–H = 100 µm. The TUNEL technique detected few apoptotic cells in the liver of animals belonging to control Groups (**I,J**) but the number increased in animals treated with CDE (**K**) and was reduced by treatment with MVC (**L**). Scale bar for I–L = 200 µm. The proliferation marker Ki67 detected few cells in control animals (**M,N**) and great numbers of positive cells in the CDE Group (**O**). The number of proliferating cells was intermediate in the CDE+MVC Group (**P**). CCR5 expression was not detected in control Groups (**Q,R**) but was found in macrophages (arrowheads), HSC (arrows), and other cell types in the CDE (**S**) and CDE+MVC (**T**) Groups. Scale bar for M–T = 100 µm.

In Groups Control and MVC the liver presented a healthy aspect, with a characteristic homogeneously rich red color. None of the livers in these Groups presented any tumor or areas of fibrosis or necrosis (Fig. 3A,B). In drastic contrast, animals in Group CDE displayed a pale yellowish liver showing a great number of tumors of different sizes, with a very hard consistency, suggesting a high degree of fibrosis (Fig. 3C). Livers belonging to mice of Group CDE+MVC were far from normal, but the number of tumors was greatly reduced when compared with Group CDE and their size was significantly smaller. In addition, tissue rigidity and general aspect of Group CDE+MVC were intermediate between Group CDE and the control Groups, Control and MVC (Fig. 3D). Moreover, the size of the gallbladder in Group CDE was much larger than those found in Group CDE+MVC and in the control Groups (Control and MVC).

### MVC Reduced Tumorigenesis at the Histological Level

To determine with detail the pathology induced by the CDE diet and the protective effects of MVC, the histology of all livers was studied. In sections stained with hematoxylin and eosin (Fig. 3E–H) a normal morphology was observed in animals of Groups Control and MVC. A normal pattern of hepatocytes separated by sinusoids, portal areas and central veins was evident. None of the samples contained areas of fibrosis, necrosis, or dysplastic processes (Fig. 3E,F). In sharp contrast, tissue samples taken from Group CDE contained large atypic hepatocytes with pleomorphic nuclei and numerous tumors. Morphological manifestations of mitosis and apoptosis were also abundant (Fig. 3G). These tumors were graded, following the Edmondson-Steiner criteria, as poorly differentiated HCC (G3), characterized by a proliferation of tumor cells in a solid or compact pattern without distinct sinusoid-like blood spaces. Neoplastic cells showed an increased nuclear/cytoplasmic ratio and frequent pleomorphism, including bizarre giant cells. Furthermore, numerous progenitor (oval) cells were observed, predominantly around portal tracts. Liver samples from Group CDE+MVC presented features that were intermediate between Group CDE and the control Groups (Control and MVC). The number of atypic cells was much lower than in Group CDE (Fig. 3H). In addition, the tumor cells were moderately differentiated (classified as G2) characterized by tumor cells arranged in a trabecular pattern, with abundant eosinophilic cytoplasm and round nuclei with distinct nucleoli, hyperchromatism, and some degree of irregularity of the nuclear membrane. In line with increased ALT levels, we found that liver sections from CDE-treated mice contained increased number of apoptotic hepatocytes (Fig. 3K) as compared with the CDE+MVC Group (Fig. 3L). In addition, liver sections of animals receiving the CDE diet showed more Ki67-positive cells (Fig. 3O) than MVC treated mice (Fig. 3P) indicating higher compensatory proliferation. The expression of CCR5 was also studied by immunohistochemistry in liver sections (Fig. 3Q–T). No detectable expression of CCR5 was found in control Groups (Fig. 3Q,R), but in animals receiving the CDE diet that expression was upregulated around portal tracks, mostly in macrophages and HSC (Fig. 3S,T).

### Quantification of Tumorigenesis, Apoptosis, and Proliferation

**Figure 4:**
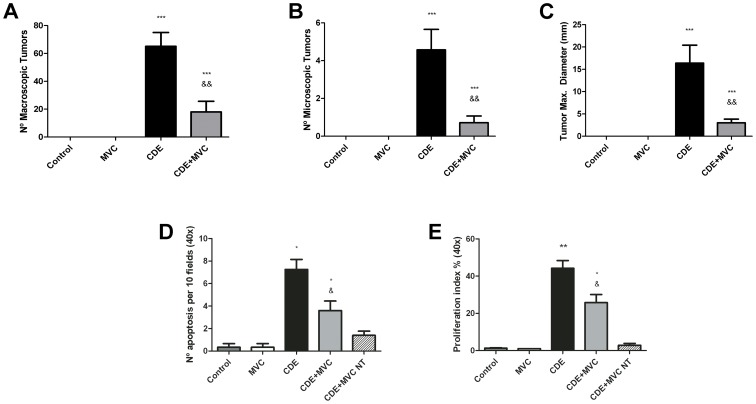
Quantification of the number of tumors, apoptotic and proliferating cells. The degree of tumor affectation was measured by counting the number of macroscopic tumors (**A**), the number of tumors seen under the microscope (**B**), and the diameter of the largest tumor on each animal (**C**). The number of apoptotic cells as determined by TUNEL (**D**), and the proliferation index, defined as the number of Ki67 positive cells divided by the total number of nuclei per field (**E**), were also quantified. The last bar, labelled “NT”, represents the non-tumoral fraction of the liver in the CDE+MVC Group. Bars represent the mean ± SEM of at least 7 animals and 5 photographs per animal (when appropriate). *p<0.05; **p<0.01; ***p<0.001 with respect to control; &p<0.05; &&p<0.01; &&&p<0.001 with respect to CDE.

To estimate the severity of the tumoral process, the number of tumors at the macroscopic and microscopic level, and the diameter of the largest tumor found in each liver were analyzed (Fig. 4A–C). Obviously, no tumors were found in animals receiving a control diet (Groups Control and MVC). In mice that received the CDE diet, there was a great difference both in the size and in the number of the tumors, being Group CDE+MVC the one presenting a lower malignancy score (p<0.01 for all parameters).

The number of apoptotic cells found in the different liver sections by TUNEL analysis was higher in the CDE Group than in the control Groups (p<0.01) but the MVC treatment significantly reduced cell death (p<0.001). Furthermore, the proliferation index, quantified from the staining with anti Ki67 antibody followed the same pattern (Fig. 4E). In the fourth experimental Group, the number of apoptotic and proliferating cells was smaller among the non tumoral parenchyma than in the tumors (Fig. 4D,E). Based on reticulin staining patterns and pathological evaluation, all liver tissue in Group CDE was considered to be tumoral, so no comparison was possible with non-tumoral tissue in this Group.

### MVC Reduced Fibrosis in Treated Mice

**Figure 5:**
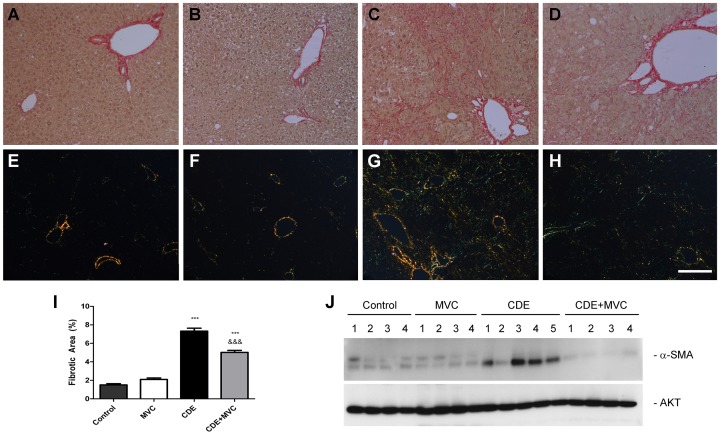
Determination of fibrosis in liver samples. Picro Sirius red staining as viewed under bright light (**A–D**) and under polarized light (**E–H**) in animals of Groups Control (**A,E**), MVC (**B,F**), CDE (**C,G**), and CDE+MVC (**D,H**). Fibrosis was widespread in animals of the CDE Group (**C,G**) and was reduced after MVC treatment (**D,H**). Scale bar for A–H = 350 µm. The fibrotic index (**I**) was calculated from the polarized light images. Bars represent the mean ± SEM of at least 7 animals and 5 photographs per animal. ***p<0.001 with respect to control; &&p<0.01; &&&p<0.001 with respect to CDE. A representative Western blot for α-SMA was performed in liver extracts from animals of the 4 experimental Groups (**J**). An antibody against AKT was used as a loading control.

To study the influence of MVC in preventing liver fibrosis, sections stained with picro-Sirius red were photographed either under bright light (Fig. 5A–D) or under polarized light (Fig. 5E–H). The areas occupied by birefringent material were quantified. As expected, animals in Group CDE presented high levels of fibrotic material whereas mice in Group CDE+MVC had significantly lower levels (Fig. 5I).

Fibrosis was also determined following the Ishak score. Groups control and MVC had no fibrosis (index 0). Group CDE had fibrous expansion of most portal areas with occasional portal to portal bridging (index 3). By contrast, Group CDE+MVC presented fibrous expansion of most portal areas, with or without short fibrous septa (index 2).

To confirm these morphological results, a Western blot was performed with liver extracts from several animals from each group and stained with an anti-α-smooth muscle actin (α-SMA) antibody (Fig. 5F). As expected, CDE animals had a much higher expression of α-SMA and the ones receiving MVC had a much reduced amount.

### MVC Treatment Reduced Liver Expression of Selected Cytokines and Chemokines

**Figure 6:**
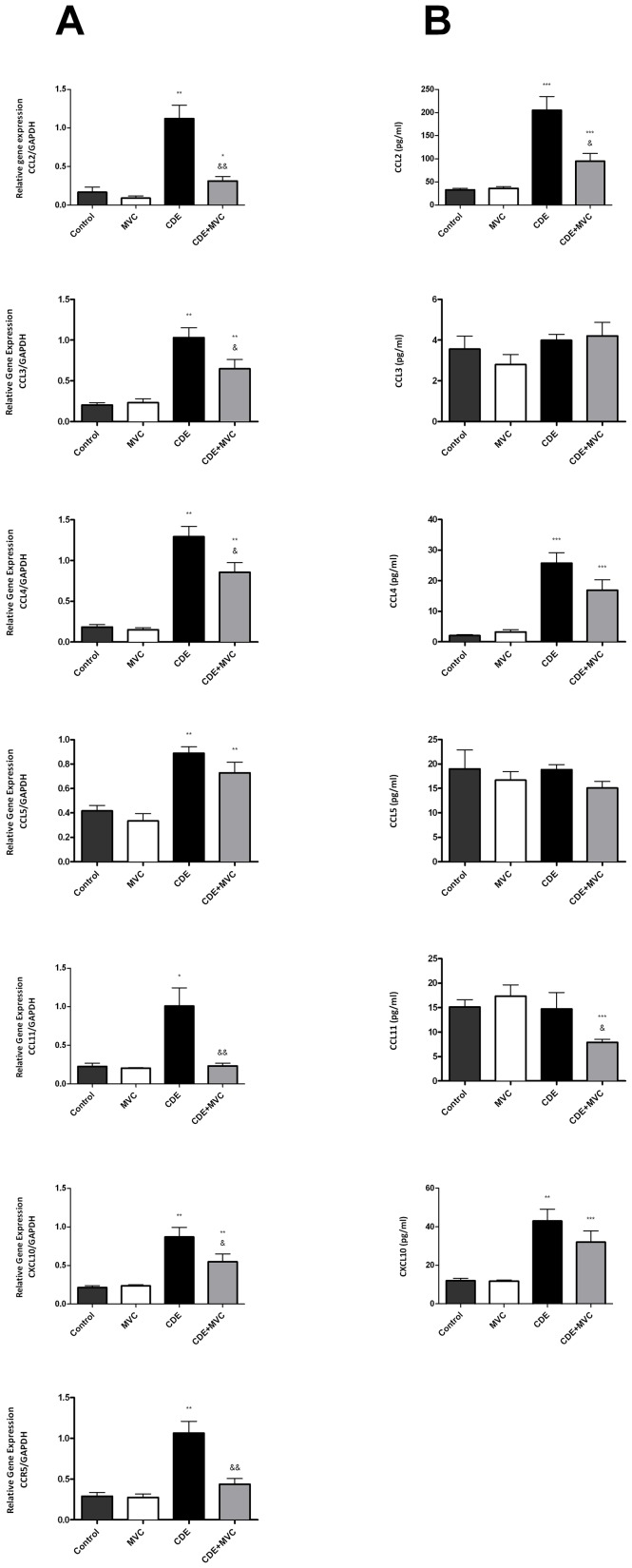
Quantification of the expression of several chemokines both at the mRNA and protein level. mRNA levels were quantified by qRT-PCR and corrected by the level of GAPDH on each sample as a house keeping gene (**A**). Proteins were analyzed by multiplex ELISA and are expressed as pg/ml (**B**). Protein assays for CCR5 were not available. Bars represent the mean ± SEM of at least 7 animals. *p<0.05; **p<0.01; ***p<0.001 with respect to control; &p<0.05; &&p<0.01 with respect to CDE.

**Figure 7:**
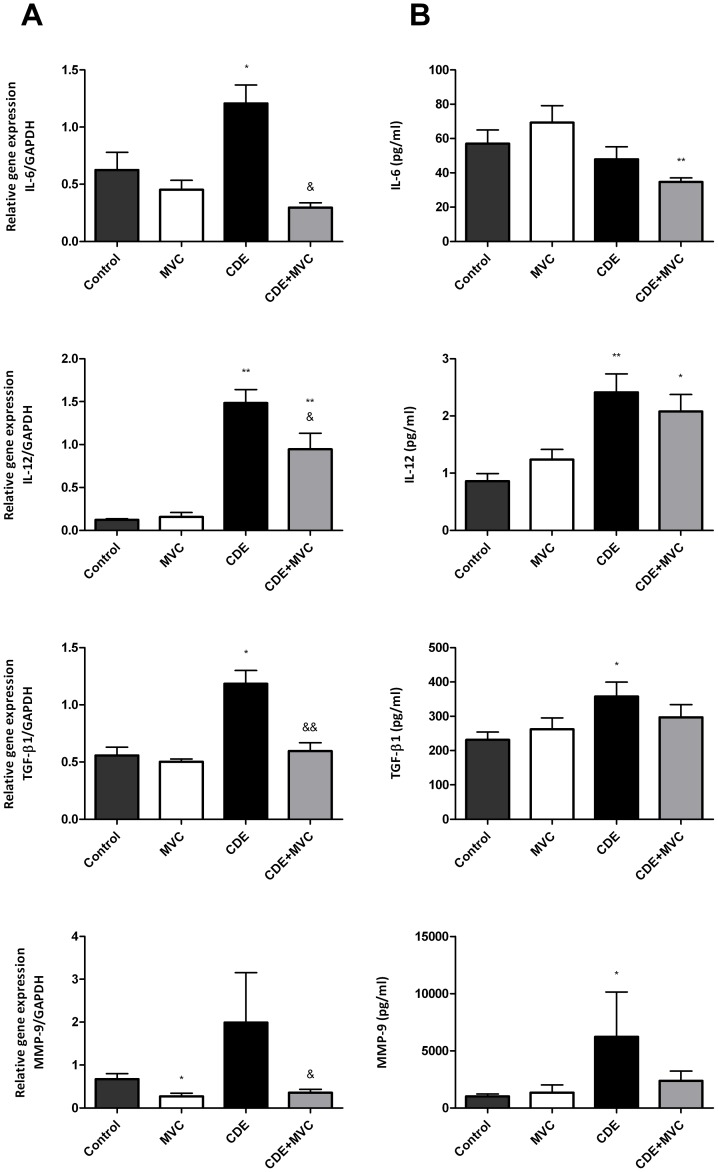
Quantification of the expression of several cytokines both at the mRNA and protein level. mRNA levels were quantified by qRT-PCR and corrected by the level of GAPDH on each sample as a house keeping gene (**A**). Proteins were analyzed by multiplex ELISA and are expressed as pg/ml (**B**). Bars represent the mean ± SEM of at least 7 animals. *p<0.05; **p<0.01; ***p<0.001 with respect to control; &p<0.05; &&p<0.01 with respect to CDE.

To better understand the mechanism underlying the previous observations, RNA and protein were extracted from liver samples and the expression of several cytokines and chemokines were quantified in the 4 experimental Groups (Figs. 6 and 7). At the mRNA level, almost all the molecules investigated showed a significant increase in animals of Group CDE and a significant correction of this increase in Group CDE+MVC (Fig. 6A, 7A). The same pattern was preserved for some molecules at the protein level. These included CCL2, CCL4, CXCL10 (Fig. 6B), IL-12, TGFβ-1, and MMP-9 (Fig. 7B). Other molecules seem to experience some kind of post-translational regulation because the protein pattern does not coincide with the RNA expression. For instance, the protein levels for CCL3 and CCL5 were similar in the 4 Groups (Fig. 6B).

### CCL5-induced Phosphorylation of p38 and ERK was Inhibited by MVC in HSC

**Figure 8:**
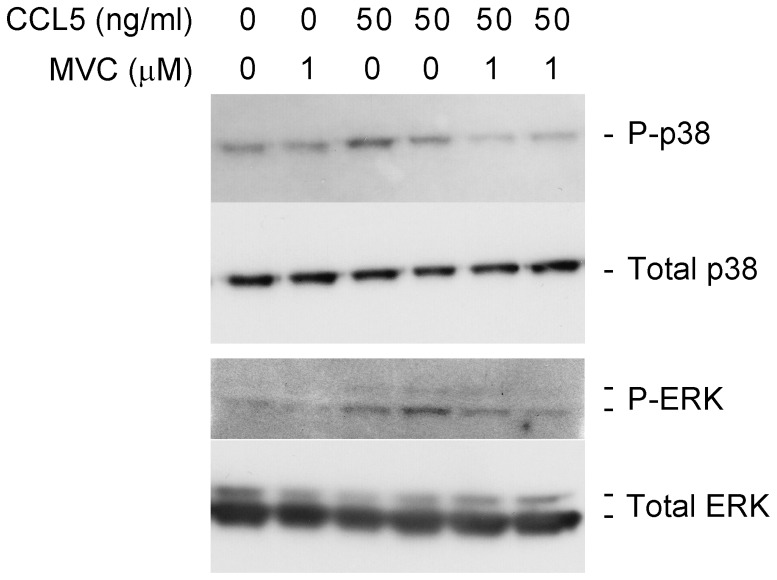
Signal transduction (p38 and ERK) in HSC. Primary human HSC were incubated with 50 ng/ml human recombinant CCL5 in the presence and absence of 1.0 µM MVC. Western blot analysis showed that CCL5 induces phosphorylation of p38 and ERK whereas preincubation of these cells with MVC prevented this activation event.

Proliferation and migration of activated HSC are considered key events in hepatic wound healing and these processes are mediated by phosphorylation of ERK (extracellular signal regulated kinase) and p38 (mitogen-activated protein kinase) respectively [13], [33]. Therefore we studied the effects of treating human HSC with human recombinant CCL5 in the presence and absence of MVC. CCL5 caused an increase in both ERK and p38 phosphorylation which was substantially attenuated by MVC (Fig. 8).

### MVC Treatment Reduces Free Radical-induced Apoptosis of Hepatocytes

**Figure 9:**
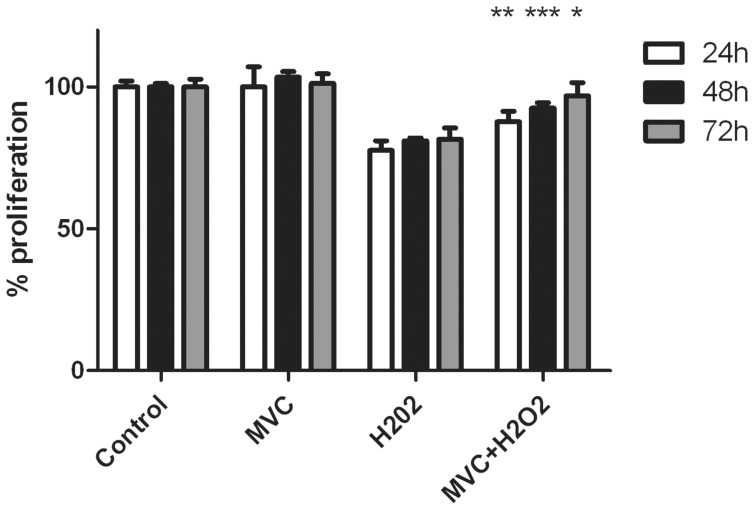
Apoptosis in hepatoma cells. Human hepatoma cell line Hep3B was exposed to H_2_O_2_ in the absence and presence of MVC for different periods of time. Hydrogen peroxide induced apoptotic cell death in this cell line, which was prevented by MVC. Bars represent the mean ± SEM of 8 independent wells. *p<0.05; **p<0.01; ***p<0.001 with respect to cells treated with H_2_O_2_ but not with MVC.

To investigate whether MVC has a direct effect on hepatocytes, these cells were exposed to H_2_O_2_ in the absence and presence of MVC. As expected, H_2_O_2_ induced cell death while MVC treatment prevented apoptosis (Fig. 9).

## Discussion

In this study we have shown that the CCR5 antagonist, MVC, was able to reduce mortality, liver fibrosis, and tumorigenesis in a mouse model of HCC. In addition, MVC diminished apoptosis and proliferation indexes, and protected liver cells from free radical-induced cell death.

No significant differences were observed for any parameter when comparing animals that received a normal diet in the presence or absence of MVC treatment. This confirms that the drug is fairly safe, as expected from a compound that has gone through clinical development and is currently used for HIV viral load suppression [34]. This safety profile is maintained even in patients with high cardiovascular risk or in those co-infected with tuberculosis or hepatitis [35].

The mechanism by which a choline-deficient, ethionine-supplemented (CDE) diet induces liver fibrosis and HCC seems to involve a direct damage to the liver parenchyma and a simultaneous blocking of hepatocyte proliferation [36]. This, in turn, induces the production of a large number of oval cells which control the remodelling of the hepatic parenchyma [37]. It has been shown that CCL5 causes the chemotaxis of liver progenitor (oval) cells [38], which may explain the large number of these cells observed in animals that had received the CDE diet.

**Figure 10:**
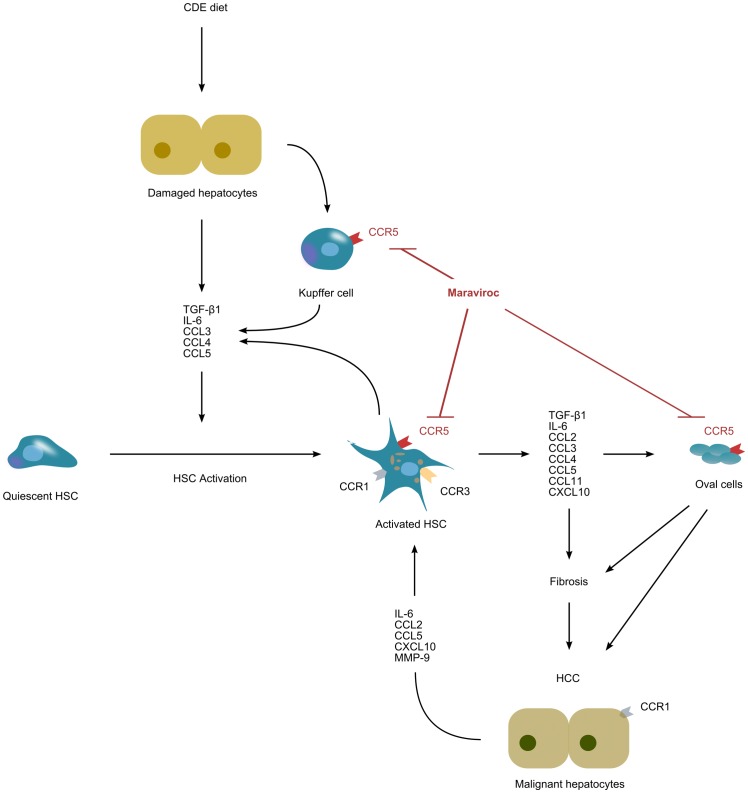
Schematic cartoon of the postulated mechanism by which MVC interferes with HCC progression. The CDE diet damages resident cells of the liver parenchyma, mainly hepatocytes, inducing oval cell proliferation. As a response, these cells and some collaborating neighbors (such as Kupffer cells) secrete a number of cytokines and chemokines including TGF-β1, CCL3, CCL4, and CCL5. The chemokine cocktail promotes the activation of HSC from a quiescent state into a more aggressive phenotype, whereupon a number of chemokine receptors (CCR1, CCR3, CCR5) are expressed at the HSC membrane. Concomitantly, activated HSC secrete a large number of chemokines and cytokines, some of which perpetuate a positive feedback loop that maintain the activated phenotype of the HSC, whereas other molecules induce fibrosis and tumor progression. Maraviroc blocks the autocrine loop by interfering with CCR5, thus stopping HSC activation, fibrosis, and tumor progression.

Damaged hepatocytes activate Kupffer cells and they collectively secrete cytokines and chemokines which trigger HSC activation [39]. This activating cocktail contains TGF-β1, IL-6, CCL3, CCL4, and CCL5, among others. All of these factors were elevated in our model when the mice received the CDE diet (Group CDE). HSC are very dynamic cells expressing various chemokine membrane receptors such as CCR1, CCR3, and CCR5 and secreting the same chemokines which are needed to maintain the activated state, indicating that the HSC are a source as well as a target of these extracellular signalling molecules [13]. Additional cells expressing CCR5 are the infiltrating T cells in injured liver, Kupffer cells [15], and liver progenitor cells, also known as oval cells [38]. This creates a reverberating positive feedback mechanism which is very difficult to suppress within the normal physiology of the liver and is the main responsible factor for the chronification of liver disease. Here is where MVC plays its major role by blocking CCR5 thus interrupting these deleterious autocrine loops (Fig. 10). There is considerable redundancy within chemokine subfamilies, with many receptors being capable of binding more than one chemokine. For instance, CCL3, CCL4, and CCL5 can bind to CCR5, but CCL3 and CCL5 can also signal through other receptors. In contrast, CCL4 activity is restricted to CCR5 binding [18]. This may explain why, at the protein level, CCL4 follows the expected pattern of rising with the CDE diet and being corrected by MVC, whereas CCL3 and CCL5 do not show such changes.

In addition to perpetuating their activated state, HSC secrete a number of fibrogenic and inflammatory chemo-cytokines. These include TGF-β1, IL-6, CCL2, and CCL5, among others. These molecules favour collagen deposition, fibrosis, and development of HCC [16], [40], [41]. All of these molecules, in our experiment, were elevated by the CDE diet and all of them where downregulated by MVC, indicating they are the main link between MVC treatment and reduction in liver fibrosis and/or HCC. The fact that all these molecules are produced by the HSC suggests that this particular cell type is the central target for the drug, as well. In addition, our *in vitro* experiments have shown that MVC blocks CCL5-induced intracellular signal transduction in HSC, further implicating this cell type in the mechanism of action of the drug.

Another positive loop appears once the HCC is established, with the malignant hepatocytes stimulating the fibrogenic actions of HSC through secretion of MMP-9, IL-6, CCL2, CCL5, and CXCL10, probably involving the NF-κB pathway [41], [42]. Since this loop includes also the HSC, MVC may be also effective in blocking HCC-induced fibrosis and HCC progression once the tumor is already present. Obviously, further research is needed to address this possibility.

Another cell type we must consider are the oval cells, also known as liver progenitor cells (Fig. 10). These cells express CCR5 [38] and can increase the number of HSC through an epithelial-mesenchymal transition process, thus contributing to liver fibrosis [43]. Moreover, oval cells may transform directly into cancer stem cells, being partly responsible for the resulting hepatocarcinoma [44], [45].

Additionally, MVC has a direct effect on protecting hepatocytes from cell damage, as demonstrated by applying free radicals to these cells in culture. High levels of free radicals are common during inflammation and ischemic states of the liver and usually lead to cellular dysfunction and cytotoxicity [46]. The efficiency of MVC in this culture setting indicates that hepatocytes may express CCR5. No clear evidence for this is found in the literature but Iser et al. show that MVC prevents HIV infection in hepatocytes, despite not being able to detect CCR5 by flow cytometry [47], suggesting that low levels of CCR5 may be present in these cells. Another CCL5 receptor, CCR1, has been reported in malignant hepatocytes [48].

CCR5-deficient mice are available and some studies have focused on the effects of this deficiency in tumor progression. Some studies have presented evidence that CCR5-deficient mice developed less lung metastases than their wild type counterparts [49], and had a reduction in intratumoral accumulation of macrophages, granulocytes, and fibroblasts, resulting in less angiogenesis [50]. In addition, CCR5- and CCR1-deficient mice have less fibrosis than their wild type littermates [15]. We need to point out that gene targeting implies the removal of a character from early development, resulting in a series of adaptive and compensatory changes in the general metabolism which makes these models difficult to compare with those relying in the treatment with a pharmacological inhibitor. From a clinical perspective, the latter would be preferable since they recapitulate better the human condition. An interesting example was the treatment of mice subjected to a different diet, deficient in methionine and choline, with Met-CCL5, a CCR5 antagonist. In this study, the authors reported inhibition of HSC activation and fibrosis regression, although they did not wait for tumor formation [18]. Concordant with our study, these authors find significant reductions in the levels of IL-6, MMP-9, and TGF-β1 when treating the animals with the antagonist, pointing to these molecules as the key regulatory factors of fibrosis that get regulated by interfering with CCR5 signalling.

In summary, we have shown that treatment with MVC, a CCR5 inhibitor, significantly reduces fibrosis and tumor load in a mouse model of HCC. These results warrant further investigation with this compound at the clinical level.

## Materials and Methods

### Ethics Statement

All procedures were carried out in accordance with the European Communities Council Directive (86/609/CEE) on animal experiments and with approval from the ethical committee on animal welfare of our institution (Comité Ético de Experimentación Animal del Centro de Investigación Biomédica de La Rioja, CEEA-CIBIR).

### Animals and Animal Model

A total of 61 male C57BL/6 mice were purchased from Charles River (Barcelona, Spain). All animals had free access to food and drink during the study.

When the animals were about 5 weeks old, they were randomly assigned to one of 4 diet Groups:

Group Control. They were fed with a choline-containing diet (No. 960414, MP Biochemicals, Illkirch, France) and tap water, n = 10.

Group MVC. The same diet as the control Group but receiving 300 mg/L maraviroc (MVC, Pfeizer, New York, NY) in the drinking water, n = 11. Mouse equivalent drug doses were calculated by using an interspecies allometric scaling factor of 12.3 to arrive to a dose for mice which is equivalent to a human dose of 300 mg/day, as previously described [51].

Group CDE. These animals received the choline-deficient diet (No. 960210, MP Biochemicals) and drinking water supplemented with 0.165% ethionine (Sigma, St Louis, MO), n = 20.

Group CDE+MVC. Animals fed with the same diet as Group CDE but receiving MVC in the drinking water at the same concentration as Group MVC, n = 20.

Mice were observed daily and all the incidences, including deaths, were recorded. In addition, animals were weighted weekly. Blood samples were collected from all surviving animals on weeks 1, 4, 8, and 16. Levels of liver damage markers (transaminases, alkaline phosphatase, and bilirubin) were measured using an automatic biochemical analyzer (Cobas C711, Roche, Madrid, Spain). All surviving animals were sacrificed on week 16. At that moment, internal organs were examined macroscopically, photographed, and weighted (liver and spleen). Macroscopic tumors were identified as whitish nodules well delimitated. These were counted and the diameter of the largest tumor per mouse was also measured. Some tissue pieces were fixed in buffered formalin for histological analysis and the rest was snap-frozen in liquid nitrogen for biochemical and molecular analyses.

### Hematoxylin-eosin and Sirius-red Staining

Following fixation, tissues were dehydrated and paraffin embedded. Tissue sections (3 µm-thick) were rehydrated and stained with hematoxylin-eosin and picro-Sirius red. The fibrotic index of each animal was calculated from polarized light microphotographs of picro-Sirius red-stained slides [52]. Three sections from different hepatic lobes were analyzed for each animal and 3 random pictures were taken from each section with the 5× objective. At least 7 animals per group were included in the analysis. The amount of birefringence was calculated with help of the ImageJ free software (The NIH, Bethesda, MD).

### TUNEL Staining

Hepatic cells undergoing apoptosis were identified by means of a TUNEL assay kit (Promega, Madison, WI), following manufacturer’s instructions. Fluorescent cells were counted and the density of cells per field was quantified with ImageJ.

### Immunohistochemical Staining

Paraffin-embedded sections were rehydrated, and antigen retrieval was performed by heating in citrate buffer (pH 6.0) for 20 min at 96°C. After blocking with normal goat serum, sections were incubated overnight with primary antibodies. These were rabbit anti-Ki67 (Master Diagnostica, Granada, Spain) at 1∶100, and rabbit anti-CCR5 (Bioss Inc., Woburn, MA) at 1∶100. The next day, following several washes in PBS, a biotinylated goat anti-rabbit (Vector, Burlingame, CA) at 1∶300 was added for 60 min, followed by the ABC complex (Vector) and developed with diaminobenzidine. Slides were counterstained with hematoxylin. Quantification of the proliferation index was performed with ImageJ.

### Western Blotting

Liver samples were lysed in mPER buffer (Thermo, Rockford, USA) that was supplemented with protease-and phosphatase inhibitors (both from Roche, Mannheim Germany). Total protein on the supernatants was calculated with the BCA kit (Pierce, Rockford, IL). Concentrated (5×) SDS sample buffer (Invitrogen) was added to each sample. Liver extracts then were boiled for 5 min and separated by electrophoresis using standard 10% SDS polyacrylamid gels (Invitrogen, Carlsbad, CA) and transferred into PVDF membranes. A mouse monoclonal antibody against α-SMA (Dako, Carpinteria, CA) was applied at a 1∶1,000 dilution overnight at 4°C. Peroxidase conjugated donkey anti-mouse antibody (Jackson Immunoresearch, Suffolk, UK) was used as the secondary antibody at a 1∶2,000 dilution. Peroxidase activity was detected with ECL reagent (GE Healthcare, Buckinghamshire, UK) and subsequent exposure to X-ray films (GE Healthcare). Membranes were stripped and reprobed with a rabbit antibody against AKT (Cell Signaling, Danvers, MA) at a 1∶1,000 concentration to confirm loading homogeneity.

### Gene Expression Quantification

Total RNA was extracted from liver samples using TRIzol (Invitrogen), purified using an RNeasy Mini kit (Qiagen, Valencia, CA), and treated with DNase I (Qiagen) following manufacturer’s instructions. cDNA was synthesized by reverse transcription of 1 µg of total RNA using the SuperScript III First-Strand Synthesis kit (Invitrogen) in a total volume of 20 µl according to the manufacturer’s instructions and was amplified by SybrGreen (Applied Biosystems, Carlsbad, CA) qRT-PCR using specific primers (Table 1) in a 7300 Real Time PCR System (Applied Biosystems). Their relative expression calculated following manufacturer’s instructions. All results were divided by their corresponding house keeping gene value.

**Table 1:** Primers used for qRT-PCR.

Name	Primer	Lengh fragment amplyfied
CCL2	Sense: 5′-GCAGTTAACGCCCCACTCA-3′	63 bp
	Antisense: 5′-CCTACTCATTGGGATCATCTTGCT-3′	
CCL3	Sense: 5′-CGTTCCTCAACCCCCATC-3′	91 bp
	Antisense: 5′-TGTCAGTTCATGACTTTGTCATCAT-3′	
CCL4	Sense: 5′-AGCCAGCTGTGGTATTCCTG-3′	150 bp
	Antisense: 5′-GAGGAGGCCTCTCCTGAAGT-3′	
CCL5	Sense: 5′-ATATGGCTCGGACACCACTC-3′	123 bp
	Antisense: 5′-GTGACAAACACGACTGCAAGA -3′	
CCR5	Sense: 5′-CGAAAACACATGGTCAAACG-3′	177 bp
	Antisense: 5′-TTCCTACTCCCAAGCTGCAT-3′	
CCL11	Sense: 5′-TCCACAGCGCTTCTATTCCT-3′	178 bp
	Antisense: 5′-CTATGGCTTTCAGGGTGCAT-3′	
CXCL10	Sense: 5′-TCCTTGTCCTCCCTAGCTCA-3′	124 bp
	Antisense: 5′-ATAACCCCTTGGGAAGATGG -3′	
IL-6	Sense: 5′-ATGGATGCTACCAAACTGGAT-3′	139 bp
	Antisense: 5′-TGAAGGACTCTGGCTTTGTCT-3′	
IL-12 p40	Sense: 5′-TTATGTTGTAGAGGTGGACTGG-3′	348 bp
	Antisense: 5′-TTTCTTTGCACCAGCCATGAGC-3′	
MMP-9	Sense: 5′-CATTCGCGTGGATAAGGAGT-3′	192 bp
	Antisense: 5′-CACTGCAGGAGGTCGTAGG-3′	
TGF-β1	Sense: 5′-GCAACATGTGGAACTCTACCAGAA-3′	106 bp
	Antisense: 5′-GACGTCAAAAGACAGCCACTCA-3′	
GAPDH	Sense: 5′-CATGTTCCAGTATGACTCCACTC-3′	136 bp
	Antisense: 5′-GGCCTCACCCCATTTGATGT-3′	
β-Actin	Sense: 5′-GGCTGTATTCCCCTCCATCG-3′	154 bp
	Antisense: 5′-CCAGTTGGTAACAATGCCATGT-3′	

Annealing temperature for all reactions was 60°C. GAPDH and β-Actin were used as housekeeping genes.

### Calculation of Protein Levels

Liver tissue samples were homogenized in lysis buffer (PBS, pH 7.4, containing Complete Protease Inhibitor Cocktail, Roche) and centrifuged to eliminate solid debris. Total protein on the supernatants was calculated with the BCA kit (Pierce) and all samples were set at the final concentration of 0.5 mg/ml. Specific protein levels were measured by the Aushon BioSystems Assay Service (Billerica, MA) using SearchLight Multiplex ELISA methodology (Aushon BioSystems). This technology allows the quantification of several proteins simultaneously from the same sample [53]. Proteins tested were CCL2, CCL3, CCL4, CCL5, CCL11, CXCL10, IL-6, IL-12, TGF-β1, and MMP-9.

### Human Stellate Cells

Isolated primary human HSC, purchased from ScienCell Research Laboratories, (Carlsbad, CA), were used between passages 2 and 6. HSC were cultured in defined medium obtained from the vendor and supplemented with 2% fetal bovine serum, penicillin (100 IU/ml), streptomycin (100 µg/ml), and stellate cell growth supplement.

After serum deprivation for 24 h, cells (1.0×10^5^ cells/well) were preincubated with 1 µM of MVC for 30 min and recombinant human CCL5 (R&D) was added at a concentration of 50 ng/ml for 15 minutes. Cellular proteins were extracted and Western blot analysis was performed as described above. Antibodies against phospho p38, total p38, phospho ERK and total ERK were all used at a dilution of 1∶1,000 (all these antibodies were obtained from Cell Signaling).

### Proliferation Assay

The human HCC cell line Hep-3B was acquired from DSMZ (Braunschweig, Germany) and cultured with MEM supplemented with 10% fetal bovine serum (Invitrogen). Cells were plated in 96-well plates at a cellular density of 10,000 cells per well in 50 µl of medium. After serum deprivation for 24 h, cells were preincubated with 1.0 µM MVC for 30 min. Hydrogen peroxide (H_2_O_2_) was added at a concentration of 5.0 µM at the indicated wells. After 24 h, all wells received 20 µl of the MTT reagent (Promega) and were incubated for 4 h at 37°C. Color intensity was measured in a plate reader at 490 nm.

### Statistical Analysis

Survival data were analyzed with the Log-rank (Mantel-Cox) and Gehan-Breslow-Wilcoxon tests. Body weight data were analyzed with ANOVA followed by the Dunnet post-hoc test. For all other data, the Kruskal-Wallis test was used followed by the Mann-Whitney U-test. All data were analyzed with GraphPad Prism 5 software and were considered statistically significant when p<0.05.
